# Nipah@20: Lessons Learned from Another Virus with Pandemic Potential

**DOI:** 10.1128/mSphere.00602-20

**Published:** 2020-07-08

**Authors:** Raúl Gómez Román, Lin-Fa Wang, Benhur Lee, Kim Halpin, Emmie de Wit, Christopher C. Broder, Mahmudur Rahman, Paul Kristiansen, Melanie Saville

**Affiliations:** a Coalition for Epidemic Preparedness and Innovations (CEPI), Oslo, Norway; b Duke-NUS Medical School, Singapore; c Icahn School of Medicine at Mount Sinai, New York, New York, USA; d Australian Centre for Disease Preparedness (ACDP), Geelong, Australia; e Laboratory of Virology, Division of Intramural Research, National Institute of Allergy and Infectious Diseases (NIAID), National Institutes of Health, Hamilton, Montana, USA; f Department of Microbiology and Immunology, Uniformed Services University, Bethesda, Maryland, USA; g Independent Consultant, Dhaka, Bangladesh; h Coalition for Epidemic Preparedness Innovations (CEPI), London, United Kingdom; University of Michigan—Ann Arbor

**Keywords:** CEPI, COVID-19, Hendra virus, Nipah virus, epidemic, henipavirus, pandemic, paramyxovirus, public health, surveillance studies, vaccines, zoonotic infections

## Abstract

Nipah disease is listed as one of the WHO priority diseases that pose the greatest public health risk due to their epidemic potential. More than 200 experts from around the world convened in Singapore last year to mark the 20th anniversary of the first Nipah virus outbreaks in Malaysia and Singapore. Most of these experts are now involved in responding to the coronavirus disease 2019 (COVID-19) pandemic. Here, members of the Organizing Committee of the 2019 Nipah Virus International Conference review highlights from the Nipah@20 Conference and reflect on key lessons learned from Nipah that could be applied to the understanding of the COVID-19 pandemic and to preparedness against future emerging infectious diseases (EIDs) of pandemic potential.

In December 2019, the Nipah Virus International Conference (Nipah@20) was cohosted by the Coalition for Epidemic Preparedness and Innovations (CEPI), World Health Organization (WHO), U.S. National Institute of Allergy and Infectious Disease (NIAID) of the National Institutes of Health (NIH), and Duke-NUS Medical School (Duke-NUS). The conference marked the 20th anniversary of the first outbreak of Nipah virus infection, an emerging infectious disease (EID) that, together with coronavirus disease 2019 (COVID-19), is currently listed as one of the WHO priority diseases (1) that pose the greatest public health risk due to their epidemic potential and the insufficient countermeasures to mitigate them.

Since its first identification in Malaysia (1998) and Singapore (1999), Nipah virus infection has caused multiple outbreaks that have thus far been limited to the Asian continent. Nipah@20 provided a forum to review the history and key scientific findings over the last 20 years and to understand the current challenges in developing Nipah diagnostics, therapeutics, and vaccines. To foster international collaboration in the context of epidemic preparedness, the conference brought together 218 scientists and public health professionals working in 21 different countries around the globe. Importantly, all henipavirus-affected countries (Australia, Bangladesh, India, Malaysia, the Philippines, and Singapore) were represented at the conference, with their delegations accounting for 46% of all attendees.

In terms of outcomes, the 2-day conference created a scientific evidence-based framework (i) to inform discussions between global health stakeholders participating in CEPI’s Joint Coordination Group (JCG) on 11 December 2019 (2), (ii) to discuss the creation of a Nipah-focused regulatory working group to facilitate data sharing and joint review of Nipah vaccine candidates, and (iii) to identify further multidisciplinary actions needed to respond to the pandemic threat posed by Nipah virus.

The proceedings from the conference are now publicly available and may be consulted using the link in reference 3. Highlights and commentaries from members of the International Organising Committee follow, with an emphasis on lessons learned from Nipah that could be applied to our understanding of the current COVID-19 pandemic and to preparedness against future emerging infectious diseases (EIDs) of pandemic potential.

### Lesson 1. Make a sound investment case for preparedness.

Nipah@20 brought together a community of scientists who, over the past 20 years, have passionately studied the epidemiology, virology, pathogenesis, and therapeutics of viruses that have pandemic potential. Many of the invited speakers were involved in understanding and responding to the 2002–2003 severe acute respiratory syndrome coronavirus 1 (SARS-CoV-1) outbreak. It is no surprise that most of these scientists are currently involved in responding to the COVID-19 pandemic. For the past 15 years, this scientific community has been heavily involved in warning governments about the possible emergence of pathogen X and about the need to invest in preparedness against such a pathogen.

In terms of policy and implementation, however, prior to the emergence of SARS-CoV-2, this scientific community had struggled to convince policy makers about the benefits to invest in research, development, and vaccine manufacturing preparedness against pandemics. As an example, even though Nipah disease has been known for more than 20 years, investment on Nipah vaccine development has been limited. The development of a sound business/investment case remains a priority to conduct advocacy and to move forward the development of effective Nipah countermeasures. CEPI has estimated that, to bring four Nipah vaccine candidates through successful phase 2a clinical trials, an investment of up to 200 million U.S. dollars (USD) would be required, assuming no risk of failure (4). This figure may seem high, but it pales in comparison to the economic and nonfinancial impact that a pandemic can create, as currently being demonstrated by COVID-19. Studies estimating the economic impact of past and present henipavirus outbreaks could provide valuable data to model a sound investment case for medical countermeasures against Nipah virus, Hendra virus, and other henipaviruses with pandemic potential. To maximize investments, the possibility of a pan-henipavirus vaccine should also be considered, similar to programs considering the development of pan-sarbecovirus vaccines (5).

### Lesson 2. Articulate how to transform surveillance in animals into impact for humans.

Regarding progress and challenges in surveillance, scientists from several countries presented their research on Nipah and Henipavirus-like sequences found in bat samples throughout Asia and Africa. In addition, the Indian Council for Medical Research (ICMR) has found several new viruses among bats and is exploring their EID potential. Opinions may vary on how to translate bat surveillance into policy and prevention of disease. However, it is clear that international collaboration and further investment on surveillance research are needed to propose policy and to prepare against potential new pathogens that will continue to emerge from bats and other animals in areas with high biodiversity (6).

### Lesson 3. Articulate diagnostics use cases and target product profiles.

In terms of Nipah diagnostics, the need for international reference standards was highlighted. Organizing and coordinating the collection of sera and virus isolates in the midst of an epidemic are of great importance for the timely establishment of standard material. International standards are needed for assay validation, calibration, comparison, and quality controls, but cost-effective diagnostic kits and devices are also needed. For example, a mobile, nucleic acid-based Nipah diagnostic system is being used and validated in India and Bangladesh, with potential applications in clinical trials and outbreak response against other EIDs. These and other methods for Nipah diagnostics were presented at the conference, together with a draft Nipah diagnostics target product profile (TPP) developed by the WHO R&D Blueprint team (7).

In the absence of an effective, Nipah-specific treatment, it could be argued that there is no clinical justification or direct clinical benefit to an individual patient when testing. However, what may not benefit the individual patient will undoubtedly benefit public health at large, and thus, save lives. Two use cases for Nipah diagnostics include rapid detection of Nipah virus infections at a peripheral health center or hospital and confirmation of active Nipah virus infection at a centralized laboratory. For the former, a TPP would envision a near-patient/point-of-care (NPT/POC) test for rapid screening; for the latter, the TPP would envision a NPT/POC for rapid confirmation in settings with higher infrastructures (8).

NPT/POC testing, contact tracing, and proactive quarantine of suspected cases are indicated at the onset of an outbreak and can quickly halt further spread, as evidenced by the Nipah outbreaks that were successfully contained by Indian authorities in Kerala in 2018 and 2019 (9). In an outbreak situation involving a novel pathogen of pandemic potential, referring to previously developed diagnostics use cases and TPPs can help to quickly articulate diagnostics strategies. In the early stages of a pandemic, widespread NPT/POC diagnostics may need to prioritize sensitivity at the expense of specificity, if needed. Widespread sensitive diagnostic testing combined with appropriate prevention and control measures has so far been the fastest way to bring the COVID-19 reproduction number (*R*_0_) to less than 1 in some countries.

### Lesson 4. Diversify the preclinical testing portfolio and create direct dialogue between lab scientists, pathologists, and health workers in the field.

Two conference sessions were dedicated to Nipah pathogenesis in animal models and to transmission and case management in humans. Data were presented on aerosol and intranasal challenges in African green monkeys (AGM), intratracheal challenge in cynomolgus macaques, and intranasal challenge in ferrets. Although models vary in recapitulating specific aspects of Nipah disease observed in humans, there was no specific mention of myocarditis observed in heart tissue. Myocarditis has been a consistent observation among Nipah virus-infected patients in India and Bangladesh, but this has not yet been reported in animal models. It is possible that this difference may be due to early humane endpoints not allowing the heart tissue from infected animals to reach the level of myocarditis deterioration observed in some Nipah virus-infected humans. There was also mention of relapse and late onset of infection observed in Malaysian patients several years after the acute infection. There is no evidence of renewed viremia during relapsed encephalitis in humans, and there is currently no animal model for relapsing Nipah disease, although many investigators have proposed such research and its importance.

For Nipah and henipaviral diseases, it is conceivable that an animal rule or similar regulatory pathway may be necessary to achieve licensure of vaccines and other countermeasures. Direct dialogue between preclinical scientists, pathologists, and health workers can better inform the design of animal models suitable for EID countermeasure development. Importantly, henipavirus research has benefited from having a diverse preclinical testing portfolio, as many different animal species are susceptible to disease, which means that preclinical studies do not have to be limited to the use of nonhuman primates (NHPs). This is also proving important for SARS-CoV-2, as research with NHPs, hamsters, mice, and ferrets ensures that COVID-19 vaccines can be tested simultaneously, according to each model, in a wider range of biosafety level 3 (BSL3) laboratory facilities for large and small animals across the world (10). It is conceivable that there will be different animal models for different endpoints such as infection, disease, and transmission. In addition, the use of human organoids is also being explored as a model to better understand SARS-CoV-2 biology in human tissue (11).

### Lesson 5. Plan for clinical trials by bringing together local and regional regulators.

Three sessions were dedicated to epidemiological needs for clinical trials and to review progress in the development of Nipah medical countermeasures. There are several therapeutics in development, including the following: lipid-anchored peptides to prevent viral fusion, low-cost compounds such as heparin, and a nasal spray with compounds for potential prophylactic use. There are also two monoclonal antibodies that can neutralize the virus and protect against Henipavirus infection and disease *in vivo*. One monoclonal antibody (m102.4) has completed a phase I trial in Australia and has been approved for compassionate use in humans (17). Since antibodies do not cross the blood-brain barrier, for monoclonal antibody-based therapeutics to be effective, virus neutralization will likely have to occur in the respiratory tract, blood, and other tissues before the virus disseminates into the brain. However, multiple Nipah challenge models including NHPs indicate that passive immunization with Nipah neutralizing antibodies can also prevent central nervous system (CNS) involvement if administered at the first signs of Nipah disease.

There was discussion on what the optimal Nipah clinical trial design would be in the context of an EID with very low incidence, i.e., in the absence of feasible randomized controlled trials (RCTs), observational studies such as case-control studies could still provide valuable evidence. Relevant clinical trials for Nipah countermeasures will require engagement of local regulatory authorities in Bangladesh (Directorate General of Drug Administration [DGDA]) and India (Drug Controller General of India [DCGI]). Partnerships are being built locally, with a focus on equitable access and low-cost interventions. Recognizing that pathways to Nipah vaccine licensure are likely to vary across countries, early dialogue between regulators from Asia, Europe, and North America was facilitated during the conference, and an international, Nipah-focused regulatory group was created. Similarly, bringing regulators together early during COVID-19 medical countermeasure development could result in the identification of actions that can be harmonized or accelerated to meet common requirements or convergences along the pathway to licensure across multiple national regulatory agencies.

### Lesson 6. Invest in diversified platform technologies that can be harnessed for speed, scale, and access during a pandemic.

One Nipah@20 session was devoted to the Nipah vaccine pipeline. At least 13 vaccine candidates have been confirmed to be under development in preclinical stages (4). Five of these active vaccine developers presented their latest preclinical data, with two speakers presenting on platform technologies that are being used for the development of COVID-19 vaccines.

Keith Chappell and Paul Young from the University of Queensland, in Brisbane, Australia, presented their molecular clamp approach to produce a highly stable prefusion form of the Nipah virus F glycoprotein, which is responsible for membrane fusion, and could be an additional target for neutralizing antibodies. The molecular clamp is a novel way of locking synthetic versions of viral surface proteins into the same shape that appears on the virus surface. This technology has been designed as a platform approach to generate vaccines against a range of human and animal viruses and has shown promising results in the laboratory targeting viruses such as influenza virus, Ebola virus, Nipah virus, and Middle East respiratory syndrome (MERS) coronavirus. This same principle is also being supported by CEPI to develop a recombinant, spike protein-based COVID-19 vaccine, with phase I trials expected to begin by July 2020.

Sarah Gilbert from the Jenner Institute, at Oxford University, United Kingdom, presented the ChAdOx1 Nipah vaccine, a simian adenovirus-based vaccine encoding Nipah virus (NiV) glycoprotein (G) Bangladesh. ChAdOx1 is a replication-deficient simian adenoviral vector which has been used for the development of vaccines against many pathogens in preclinical and clinical studies. It is safe for use in all ages and in patients who are immunocompromised, inducing strong and well-maintained humoral and T cell responses against the encoded antigen without the requirement for an adjuvant. A single dose of a ChAdOx1-vectored Nipah vaccine protects against homologous (Bangladesh strain) and heterologous (Malaysia strain) challenge in the intraperitoneal Syrian golden hamster challenge model (12). Sarah Gilbert’s team is now using the same chimpanzee adenoviral platform technology to develop a COVID-19 vaccine candidate, supported in part by CEPI. A single dose of the vaccine, which encodes the spike protein of SARS-CoV-2, may confer protection against disease in a rhesus macaque pneumonia model (13). This COVID-19 vaccine candidate has now entered phase I/II trials in the United Kingdom (EU Clinical Trials Register [https://www.clinicaltrialsregister.eu/ctr-search/trial/2020-001072-15/GB]), thus becoming the first CEPI-supported vaccine technology platform to move from pathogen sequence availability to phase I trials in less than 18 weeks.

Investment in many other vaccine platform technologies has resulted in an unprecedented speed, moving quickly from pathogen sequence availability in early 2020 to a diverse portfolio of more than 70 vaccine candidates in less than 4 months (14). Although regulatory agencies are adapting to keep up with such a rapidly evolving vaccine pipeline, preparedness for future pandemics may necessitate regulatory frameworks to preapprove specific platforms with consistent safety data in order to save precious time when plugging in a novel pathogen sequence into a vaccine platform technology. For many COVID-19 platform technologies, dialogue is ongoing to articulate strategies for large-scale manufacturing (15) and access (16).

### Lesson 7. Engage regionally and locally, not just globally.

The final conference session was dedicated to collaboration and synergy. Zhengli Shi, from the Wuhan Institute of Virology/Chinese Academy of Sciences (CAS), described how a Sino-French agreement to collaborate on EIDs was established in 2004, following the SARS-CoV outbreak. BSL4 labs were established in the Wuhan Institute of Virology as a result of the SARS-CoV outbreak. Prior to the COVID-19 pandemic, the institute had an annual call launched around April each year for external organizations wanting access to the facility to test their own samples or to have access to samples from the in-house repositories. The many years of research experience and international collaborations built by the CAS since 2004 have provided a preparedness framework through which Chinese scientists have responded and continue to respond to the SARS-CoV-2 epidemic 16 years later.

Nivedita Gupta, from the Indian Council of Medical Research (ICMR), described the Regional Enabler for the Southeast Asia Research Collaboration for Health (RESEARCH) platform. The RESEARCH platform was established on 28 August 2019, with ICMR as the secretariat. The goal of the platform is to facilitate clinical research across the region, leveraging the unique capabilities of each country and sharing expertise. Of the 11 member states in the WHO Southeast Asia Region, 10 participated in the second RESEARCH platform meeting. With the platform in place, it will be easier to mount rapid responses to new outbreaks and to conduct clinical trials—not only on Nipah virus, but also other EIDs.

### Epilogue.

Unbeknownst to the scientists attending the Nipah@20 conference in Singapore in December 2019, the COVID-19 outbreak had already begun. We started the year 2020 confronted with a new EID that required rapid mobilization for vaccine development. Nipah@20 was a scientific drill, a timely dialogue about the need to invest in the development of vaccines and medical countermeasures against EIDs with pandemic potential. CEPI has since mobilized early investment for several COVID-19 vaccine candidates. As stated earlier, for two awardees, this builds on work already undertaken with Nipah virus vaccine projects which were showcased at the Nipah@20 conference.

Transparency and a collaborative spirit underpinned the Nipah@20 conference. Although Nipah virus may not seem like a priority in the current COVID-19 context, we hope that the scientific momentum will continue, with high-value EID-related research being undertaken and published. When will Henipavirus experts next meet? An unofficial suggestion has been to commemorate the 30th anniversary of the discovery of Hendra virus, which was the founding member of the Henipavirus genus, sometime in 2024.
